# Targeting Heat Shock Transcription Factor 4 Enhances the Efficacy of Cabozantinib and Immune Checkpoint Inhibitors in Renal Cell Carcinoma

**DOI:** 10.3390/ijms26041776

**Published:** 2025-02-19

**Authors:** Saeki Saito, Hirofumi Yoshino, Seiya Yokoyama, Mitsuhiko Tominaga, Gang Li, Junya Arima, Ichiro Kawahara, Ikumi Fukuda, Akihiko Mitsuke, Takashi Sakaguchi, Satoru Inoguchi, Ryosuke Matsushita, Yasutoshi Yamada, Shuichi Tatarano, Akihide Tanimoto, Hideki Enokida

**Affiliations:** 1 Department of Urology, Graduate School of Medical and Dental Sciences, Kagoshima University, Kagoshima 890-8520, Japan; kendo_3110@yahoo.co.jp (S.S.); gatyamanbo_win@yahoo.co.jp (M.T.); happy-lg@outlook.com (G.L.); j.arima.with.bassoon@gmail.com (J.A.); kawaharaichiro1@icloud.com (I.K.); fukuda.i@m.kufm.kagoshima-u.ac.jp (I.F.); aki.lefty8@gmail.com (A.M.); sg-t1628@m.kufm.kagoshima-u.ac.jp (T.S.); k6897847@kadai.jp (S.I.); k9415138@kadai.jp (R.M.); k9876942@kadai.jp (Y.Y.); taracho@m3.kufm.kagoshima-u.ac.jp (S.T.); henokida@m2.kufm.kagoshima-u.ac.jp (H.E.); 2 Department of Pathology, Graduate School of Medical and Dental Sciences, Kagoshima University, Kagoshima 890-8544, Japan; yokoyama@m3.kufm.kagoshima-u.ac.jp (S.Y.); akit09@m3.kufm (A.T.)

**Keywords:** cabozantinib, heat shock transcription factor 4, immune checkpoint inhibitor, MET, renal cell carcinoma

## Abstract

Recently, immune checkpoint inhibitors (ICIs) and cabozantinib, a tyrosine kinase inhibitor (TKI), have been used to treat renal cell carcinoma (RCC); the combination of these agents has become a standard treatment for RCC. TKIs generally target vascular endothelial growth factor. However, cabozantinib is characterized by its targeting of MET. Therefore, cabozantinib can be used as a late-line therapy for TKI-resistant RCC. According to data from The Cancer Genome Atlas (TCGA), heat shock transcription factor 4 (*HSF4*) expression is higher in RCC tissues than in normal renal tissues. HSF4 binds to the MET promoter in colorectal carcinoma to enhance MET expression and promote tumor progression. However, the functional role of HSF4 in RCC is unclear. We performed loss-of-function assays of *HSF4*, and our results showed that *HSF4* knockdown in RCC cells significantly decreased cell functions. Moreover, MET expression was decreased in *HSF4*-knockdown cells but elevated in sunitinib-resistant RCC cells. The combination of cabozantinib and *HSF4* knockdown reduced cell proliferation in sunitinib-resistant cells more than each monotherapy alone. Furthermore, *HSF4* knockdown combined with an ICI showed synergistic suppression of tumor growth in vivo. Overall, our strategy involving *HSF4* knockdown may enhance the efficacy of existing therapies, such as cabozantinib and ICIs.

## 1. Introduction

Renal cell carcinoma (RCC) constitutes approximately 90% of all renal malignancies and represents the 14th most frequently diagnosed cancer, with more than 400,000 new cases worldwide in 2020 [1,2]. Clear-cell RCC is the most common subtype of kidney cancer, accounting for approximately 80% of cases, followed by papillary RCC, which occurs in approximately 10–16% of cases [3]. Many patients (37–61%) are diagnosed with RCC incidentally based on abdominal imaging studies, such as ultrasound or computed tomography. Approximately 70% of patients are diagnosed with stage I RCC (with renal masses less than 7 cm in size), and 11% of patients are diagnosed with stage IV disease [4].

Surgery, immune checkpoint inhibitors (ICIs), tyrosine kinase inhibitors (TKIs), and combinations of these therapies are currently used to treat patients with RCC according to the disease stage. For patients with stage I disease, partial nephrectomy can result in a 5-year cancer-specific survival of more than 94%. For advanced or metastatic RCC, combinations of ICIs with TKIs are associated with a tumor response of 42–71% and a median overall survival (OS) of 46–56 months [4].

Despite the emergence of several different therapeutic drugs, not all patients with RCC show good responses to therapy. Although many studies are investigating how to overcome drug resistance, the search for new therapeutic targets remains challenging. While there are several known genetic alterations associated with RCC, such as the mutation of VHL, we have attempted to search for new target genes using The Cancer Genome Atlas (TCGA) database [5]. TCGA is a project initiated in the United States in 2006 as part of the Cancer Genome Project, which has yielded important data on cancer diagnosis, treatment, and prevention; these data are now available to researchers [6]. Using this database, it is possible to easily examine gene expression and its impact on prognosis based on clinical information. However, the sheer volume of data requires advanced statistical knowledge and skills to perform analyses.

In this study, we performed a comprehensive analysis of TCGA data on RCC and listed genes that were highly expressed in RCC and were associated with a significantly poorer prognosis in the high expression group in prognostic analysis. From this analysis, we identified multiple genes that had not been frequently reported in RCC. The purpose of this study was to explore uncharacterized genes in RCC and to elucidate the cancer signaling pathways affected by these genes in order to provide basic data for new therapeutic strategies and to propose new therapeutic possibilities in RCC.

Notably, we detected heat shock transcription factor 4 (*HSF4*) as a prognostic gene in patients with RCC based on data from TCGA. HSF4 has been reported to promote tumor progression by enhancing MET expression in colorectal carcinoma cells [7]. Several drugs have been shown to target MET expression; however, while savolitinib is currently in clinical trials for papillary RCC [8], cabozantinib has only been approved as the MET targeting TKI for treatment of RCC. Cabozantinib is used not only as a single agent but also in combination with an ICI, contributing significantly to the improvement of RCC outcomes. Because cabozantinib is also used as a late-line therapy against TKI-resistant RCC, we investigated MET expression and the therapeutic effects of cabozantinib and *HSF4* knockdown in sunitinib-resistant RCC previously studied in our department [9]. In combination therapy of a TKI and ICI, the TKI has been shown to enhance the tumor-suppressive effect of the ICI by inhibiting the immunosuppressive roles of vascular endothelial growth factor receptor (VEGFR) and MET [10]. Therefore, we focused on the association between HSF4, which regulates MET expression, and an ICI to investigate the effects of combination therapy.

In this study, we first searched for genes associated with poor prognosis in patients with RCC using data from TCGA. Next, the effects of knockdown of the target gene were examined by cell function analysis and RNA sequencing. Finally, we investigated the relationship between the target gene and standard therapies for RCC, such as cabozantinib and ICIs.

## 2. Results

### 2.1. Expression Levels of HSF4 in RCC Tissue and Normal Renal Tissue

**Figure 1:**
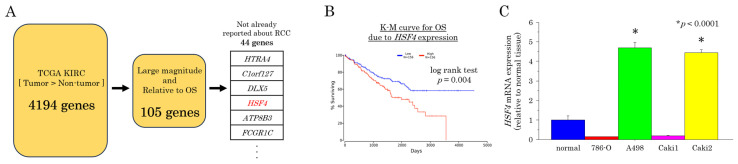
Expression levels of *HSF4* in RCC tissue and normal renal tissue. (**A**) In silico analysis of mRNA expression levels in the KIRC group from TCGA data suggested that *HSF4* was a good target gene for our experiments. Mann–Whitney *U* tests were used to test differences between the two groups. Cliff’s delta was calculated for the effect size test. The analyses were conducted using R programming language version 4.4.2. (**B**) Overall survival in renal clear-cell carcinoma (KIRC) cohorts based on TCGA data. *HSF4* expression was compared with that in normal samples (n = 156; Mann–Whitney *U* test). (**C**) *HSF4* mRNA expression levels were compared using RT-qPCR of total RNA samples from normal kidneys and total RNA extracted from the human RCC cell lines 786-O, A498, Caki1, and Caki2. Each experiment was repeated at least three times. The Bonferroni/Dunn method was used as a multiple comparisons test.

To search for new targets for the treatment of RCC, we used the KIRC group in TCGA database. We chose the KIRC group because it is a cohort composed of patients with clear-cell carcinoma, the most representative histologic type of RCC, and has a large sample size. We identified 4194 genes that were upregulated more in RCC tissues than in normal renal tissues. Among these 4194 genes, those with large effect sizes in terms of differences in expression and those associated with poor prognosis (poor OS) were selected and narrowed down to 105 genes. In addition, 44 genes were found to have no previously reported association with the kidney; *HSF4* was in the top group on that list (Figure 1A). Because the other top genes were less frequently expressed in normal renal tissues, we focused our study on HSF4. HSF4 has been reported to be involved in eye lens formation [11], but its role in RCC is unclear. In patients with RCC, OS was significantly shorter in the high-*HSF4*-expression group than in the low-*HSF4*-expression group (n = 156; *p* = 0.004, Figure 1B). We compared the expression levels of *HSF4* in total RNA samples from the normal kidney and total RNA extracted from the human RCC cell lines 786-O, A498, Caki1, and Caki2 using reverse transcription quantitative polymerase chain reaction (RT-qPCR). *HSF4* expression was significantly higher in A498 and Caki2 cells than in normal renal tissues (Figure 1C). Based on these results, we decided to use A498 and Caki2 cells in subsequent experiments.

### 2.2. Effects of HSF4 Knockdown Using Small Interfering RNA (siRNA) in RCC Cells

**Figure 2:**
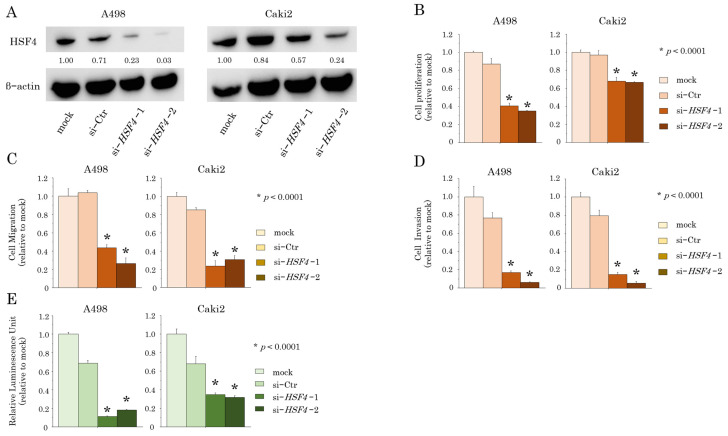
The effects of *HSF4* knockdown using si-*HSF4* in RCC cells. (**A**) HSF4 protein expression levels were decreased in *HSF4*-knockdown A498 and Caki2 cells, as determined by Western blotting. The values listed are in comparison with β-actin expression. (**B**) Cell proliferation measured using XTT assays. (**C**) Cell migration was measured using wound healing assays. (**D**) Cell invasion was evaluated using Matrigel invasion assays. Infiltrating cells were counted and compared with parental and *HSF4*-knockdown RCC cells. (**E**) Tumor mass formation was measured using tumor spheroid formation assays. The percentage of viable cells was measured as luminosity in proportion with ATP production. Each of the experiments was repeated at least three times. In all experiments, si-*HSF4* transfectants were compared with mock transfection. The Bonferroni/Dunn method was used as a multiple comparisons test.

To investigate the role of HSF4 in RCC, we performed *HSF4* knockdown using siRNA in A498 and Caki2 RCC cells. Two siRNAs significantly knocked down *HSF4* at both the mRNA and protein levels, as confirmed by RT-qPCR and Western blotting (Figure 2A and Figure S1A). In XTT assays, the proliferative abilities of *HSF4*-knockdown RCC cells were markedly decreased compared with those of the parental cell lines (Figure 2B). In wound healing assays and Matrigel invasion assays, migration and invasion abilities were significantly decreased in *HSF4*-knockdown cells compared with those in the parental cell lines (Figure 2C,D and Figure S1B,C). Furthermore, in tumor spheroid formation assays, the tumor mass formation abilities of *HSF4*-knockdown cells were significantly decreased compared with those of the parental cell lines (Figure 2E and Figure S1D). These results suggested that the knockdown of *HSF4* decreased the viability of RCC cells.

### 2.3. Relationship Between HSF4 and Cell Apoptosis

**Figure 3:**
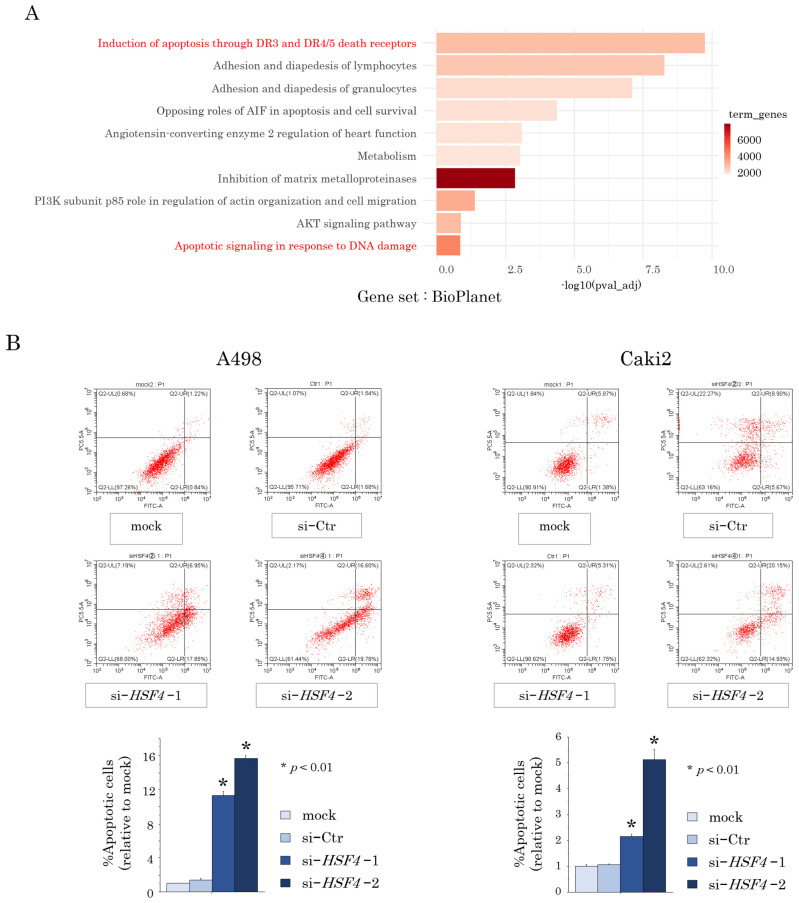

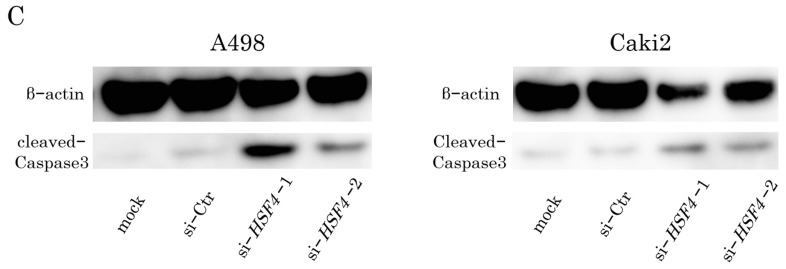
RNA sequencing analysis and cell apoptosis assays. (**A**) RNA sequencing was performed using total RNA extracts from *HSF4*-knockdown RCC cells and parental cell lines to compare gene expression. Gene set analysis was performed using the BioPlanet gene set in GeneCordis 4. (**B**) Flow cytometry was used to assess the percentages of apoptotic cells among RCC cells transfected with si-*HSF4* or si-Control (Ctr). Each of the experiments was repeated at least three times. The Bonferroni/Dunn method was used as a multiple comparisons test. (**C**) Western blotting analysis was performed to compare the expression levels of cleaved caspase 3 in *HSF4*-knockdown RCC cells and the parental cell lines.

To further investigate the role of HSF4 in RCC, we performed RNA sequencing using total RNA extracted from parental and *HSF4*-knockdown A498 and Caki2 cells. Analysis of the sequencing results using the BioPlanet dataset revealed significant differences in the expression of several gene groups related to apoptosis between the *HSF4*-knockdown RCC cells and the parental cell lines (Figure 3A). Based on these results, we performed cell apoptosis assays using the flow cytometer. The results showed that the ratios of apoptotic cells were significantly increased following *HSF4* knockdown in both A498 and Caki2 cell lines (Figure 3B). In addition, Western blot analysis showed that the expression of cleaved caspase 3, a protein associated with apoptosis, was significantly elevated in *HSF4*-knockdown cells (Figure 3C). Thus, the RNA sequencing results were confirmed by these experiments.

### 2.4. HSF4 Regulated MET Expression and Promoted Tumor Progression in RCC Cells

**Figure 4:**
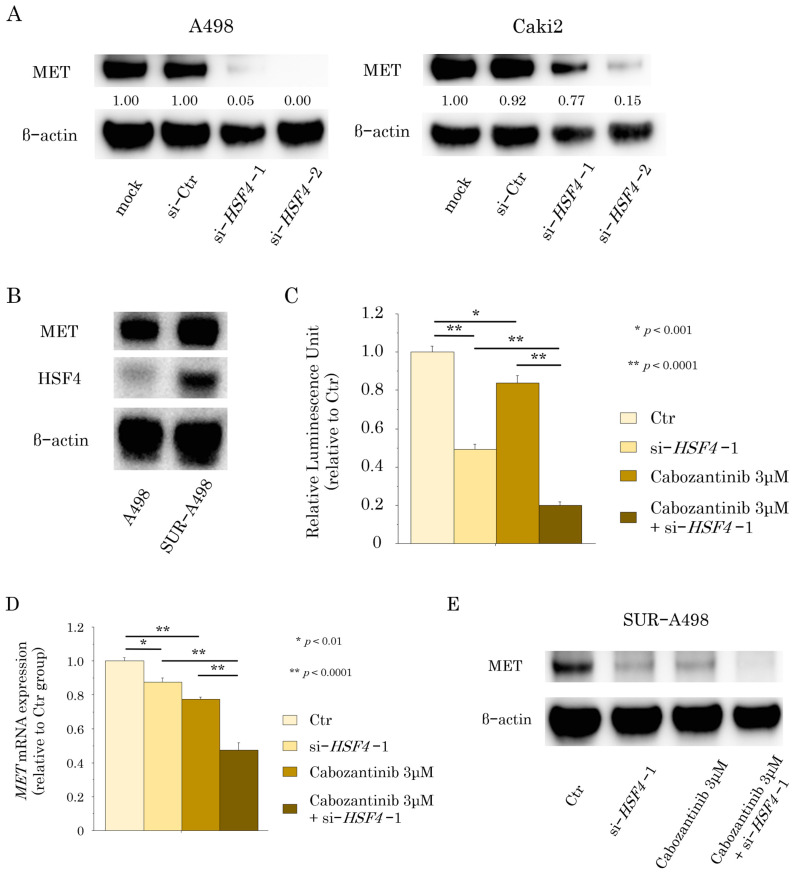
HSF4 regulated MET expression and promoted tumor progression in RCC cells. (**A**) Western blotting analysis of MET expression in *HSF4*-knockdown RCC cells and the parental cell lines. (**B**) Western blotting analysis of MET and HSF4 expression in sunitinib-resistant RCC cells and the parental cells. (**C**) Cell proliferation assays were performed on sunitinib-resistant RCC cells using si-*HSF4*, si-Ctr, and cabozantinib. The percentages of viable cells were measured as luminosity in proportion to ATP production. The Bonferroni/Dunn method was used as a multiple comparisons test. The experiment was repeated at least three times. (**D**) *MET* mRNA expression levels were compared using RT-qPCR of total RNA extracted from cells in each group. Each experiment was repeated at least three times. The Bonferroni/Dunn method was used as a multiple comparisons test. (**E**) Western blotting analysis of MET expression in cells in each group.

HSF4 directly binds to the MET promoter and enhances its expression, thereby promoting tumor progression in colorectal carcinoma [7]. Therefore, we focused on the association between HSF4 and MET in RCC. Our results showed that MET was significantly downregulated in *HSF4*-knockdown RCC cells compared with that in the parental cell lines (Figure 4A), suggesting that HSF4 regulated MET expression in RCC. However, MET expression was upregulated in sunitinib-resistant A498 cells (SUR-A498 cells) previously established in our laboratory compared with that in the parental cell line (Figure 4B) [12]. Moreover, HSF4 expression was also upregulated in SUR-A498 cells (Figure 4B). Because the TKI cabozantinib targets MET, we investigated the effects of HSF4 knockdown and cabozantinib in SUR-A498 cells. The concentration of cabozantinib was determined based on the IC50 in SUR-A498 (Supplementary Figure S2). *HSF4* knockdown or cabozantinib treatment inhibited SUR-A498 cell proliferation, and combination treatment with both *HSF4* knockdown and cabozantinib enhanced this inhibitory effect on cell proliferation (Figure 4C). The inhibitory effects of each group on cell proliferation reflected the inhibition of MET expression (Figure 4D,E). The inhibitory effect of the combination therapy was evaluated using the Bliss independence model and determined to be synergistic (Supplementary Table S1).

### 2.5. HSF4 Knockdown Inhibited Tumor Progression In Vivo When Combined with an ICI

**Figure 5:**
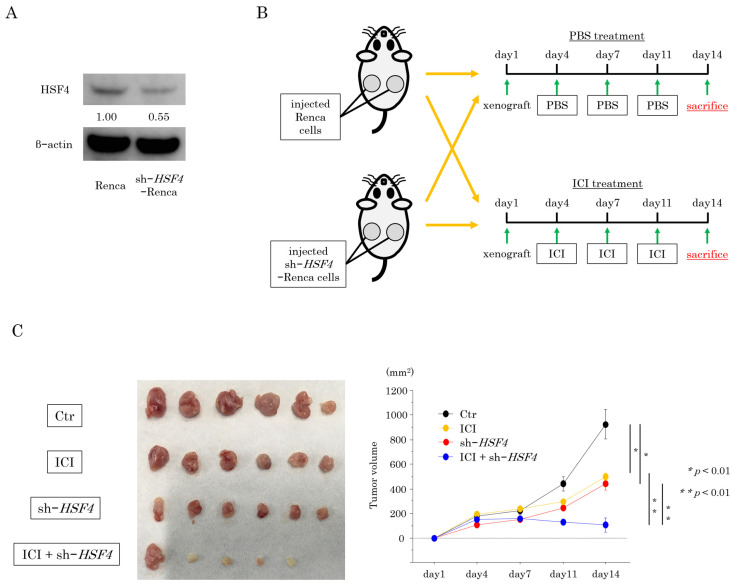

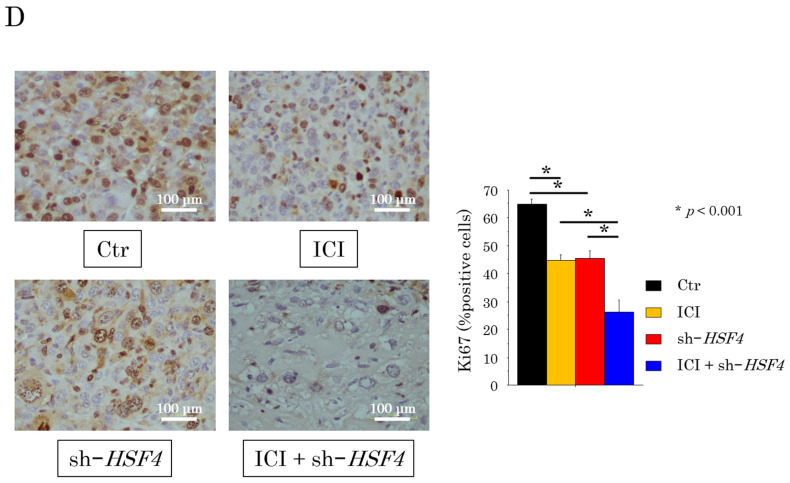
Generation of shRNA-transfected Renca cells and xenograft assays in mice. (**A**) HSF4 protein expression levels were decreased in HSF4-knockdown Renca cells, as determined by Western blotting. (**B**) The animal model was set up as follows (n = 3 mice per group): group 1, mice injected with parental Renca cells and administered PBS; group 2, mice injected with parental Renca cells and administered the ICI; group 3, mice injected with *HSF4*-knockdown Renca cells and administered PBS; and group 4, mice injected with *HSF4*-knockdown Renca cells and administered the ICI. (**C**) Comparison of tumor volumes in mice. The Bonferroni/Dunn method was used as a multiple comparisons test. (**D**) Photograph showing Ki67-positive cells in the excised tumor stained using immunohistochemistry (400×). The percentages of Ki67-positive cells were analyzed using the Bonferroni/Dunn method.

To further investigate the effects of *HSF4* knockdown in RCC, we transfected Renca cells with short hairpin RNA (shRNA) to generate *HSF4*-knockdown cells (Figure 5A). These cells were then used in xenograft assays. Half of the mice were subcutaneously injected with *HSF4*-knockdown Renca cells, and the other half were injected with parental Renca cells (Figure 5B). Each group was further divided into two groups (n = 3 mice per group) and allocated to receive phosphate-buffered saline (PBS) or ICI by intraperitoneal injection twice a week (Figure 5B). When tumors were removed 2 weeks later, tumor growth was significantly suppressed in the group with the combination of *HSF4* knockdown and ICI treatment compared with each treatment alone (Figure 5C, Supplementary Table S2A). The suppressive effect of the combination therapy was evaluated using the Bliss independence model and determined to be synergistic (Supplementary Table S2B). No significant differences were observed in the body weights of mice in each group (Supplementary Figure S3). Immunohistochemistry showed that Ki67 expression was decreased to a greater extent in the combination group than in the groups with *HSF4* knockdown alone or ICI treatment alone (Figure 5D).

## 3. Discussion

Our research has focused on elucidation of the mechanisms of drug resistance and overcoming drug resistance in RCC. For example, we reported that Rapalink-1, a next-generation mammalian target of rapamycin inhibitor, has greater tumor-suppressive effects than temsirolimus against sunitinib-resistant RCC cells [12] and that SCG2 regulates angiogenesis in sunitinib-resistant RCC via the VHL/hypoxia-inducible factor/VEGF pathway [13]. Although target gene searches have been conducted using RNA sequencing and other methods, the complexity of the resistance mechanism and the large number of genes involved, as well as the lack of comprehensive analyses of large clinical data on RCC in our laboratory, have posed challenges in the search for therapeutic target genes. In fact, when we searched for genes involved in sunitinib resistance in our previous report, we identified *SCG2* using RNA sequencing of sunitinib-resistant strains; however, in the current study, we incorporated prognostic analysis as well as comprehensive expression analysis using TCGA data. In doing so, we were able to identify *HSF4* as an important gene in RCC.

HSFs are widely known as master regulators of the heat shock response. By contrast, HSF4 is primarily known for its involvement in tissue development [14], especially associated with cataracts, as demonstrated in multiple studies [15,16,17]. There are two isoforms of HSF4, HSF4a and HSF4b, and these differences occur during the RNA splicing phase. HSF4a has been reported to act as an inhibitor of heat shock gene expression, while HSF4b acts primarily as a transcriptional activator [18,19]. It is not clear how the two isoforms of HSF4 behave in renal tissue. Several reports have also described the association between HSF4 and some types of carcinoma. In addition to the relationships between HSF4 and colorectal carcinoma mentioned above, HSF4 has been reported to be associated with the progression of prostate cancer [20], pancreatic cancer [21], and hepatocellular carcinoma [22]. HSF4 has already been reported to act in an inhibitory manner against apoptosis in colon cancer cells and lens formation, and our experiment revealed that it does the same in RCC by RNA sequencing and flow cytometry [23,24]. Furthermore, HSF4 has been shown to be involved in the MET [7], extracellular signal-regulated kinase [25], and AKT [22] pathways in cancer. However, this is the first report to describe the association between RCC and HSF4.

In this study, we showed that *HSF4* knockdown significantly reduced the proliferative, migratory, and invasive potential of RCC cells. We also found that HSF4 regulated MET expression in RCC, consistent with a previous report describing this interaction in colorectal carcinoma [7]. The *MET* oncogene is located on chromosome 7p21-31, and its protein product is a MET receptor-type tyrosine kinase [26]. This receptor appears in the epithelial cells of many organs, including the liver, pancreas, prostate, kidney, muscle, and bone marrow, both in embryonic development and in adulthood [27]. MET and its ligand, hepatocyte growth factor (HGF), are observed in most solid tumors, and signaling via MET is known to be associated with a number of malignant tumors [28]. When HGF binds to MET, it affects tumors through a variety of signaling pathways, including those involved in tumorigenesis, cell proliferation, cell motility, and cell cycle progression [29,30]. When overexpressed by mutation or amplification, MET is abnormally activated in cancer [26]. Previous reports have shown associations between MET overexpression and many types of cancers, including colorectal carcinoma, ovarian cancer, and lung adenocarcinoma [31,32,33]. In the field of urology, cabozantinib has been used as a treatment for advanced RCC. Unlike previously used TKIs, such as sunitinib, pazopanib, and axitinib, cabozantinib targets MET in addition to VEGFR and may be effective as a treatment for RCC cells that are resistant to other TKIs [34]. Notably, we found that the combination of cabozantinib and *HSF4* knockdown enhanced the efficacy of cabozantinib in sunitinib-resistant RCC cells. We expected it to be an additive effect since the two drugs also target MET, but in fact the result suggested that it was a synergistic effect. Further studies in cabozantinib-resistant RCC cells are needed to assess whether *HSF4* knockdown can overcome cabozantinib resistance as well.

The development of ICIs has revolutionized the treatment of RCC. Indeed, ICIs are now becoming the standard of treatment for advanced RCC. There is a wide range of treatment options, including multiple ICIs and combination therapy with TKIs [35]. In combination treatments using TKIs and ICIs, TKIs have been shown to enhance the effects of ICIs by inhibiting the immunosuppressive roles of VEGFR and MET [10]. In this study, we showed that an ICI exerted stronger tumor-suppressive effects when used in combination with *HSF4* knockdown in vivo. Although there are no previous reports showing a direct association between HSF4 and immune checkpoints, the tumor-suppressive effect of *HSF4* knockdown in combination with an ICI was considered to be a synergistic response acting through correction of the immunosuppressive environment via MET inhibition. Further research is needed to confirm this mechanism; however, the results of our experiment highlighted the potential applications of this novel combination therapy using an ICI. In addition, shRNA was used to suppress *HSF4* expression in our animal studies because no inhibitors against HSF4 have been reported. Thus, further studies are needed to develop HSF4 inhibitors and RNAi in the future.

In summary, we identified *HSF4* as a prognostic gene in RCC using TCGA data. *HSF4* knockdown regulated MET expression and significantly reduced cell proliferation, migration, and invasion in RCC. RNA sequencing also revealed increased apoptosis in *HSF4*-knockdown RCC. In addition, *HSF4* knockdown was found to potentiate the effects of existing therapies, such as cabozantinib and ICIs. When RNAi, for example, siRNA, is used as a therapeutic agent, off-target effects and RNA instability are problems, and specific delivery to target organs is necessary. Several studies on siRNA using chemical modifications and other methods have been reported, and clinical applications of RNAi are expected [36,37,38]. Although our study had some limitations that need to be addressed for clinical application of the findings, the combination treatment identified in this study may be a promising candidate for the management of RCC.

## 4. Materials and Methods

### 4.1. Renal Cell Carcinoma Cell Lines and Cultures

The human RCC cell lines 786-O, A498, Caki1, and Caki2 and the mouse RCC cell line Renca were obtained from the American Type Culture Collection (Manassas, VA, USA). In addition, for some experiments, we used sunitinib-resistant A498 (SUR-A498) cells previously established in our laboratory. These RCC cell lines were cultured in RPMI 1640 medium supplemented with 10% fetal bovine serum at 37 °C in an atmosphere containing 5% CO_2_.

### 4.2. In Silico Analysis

Data for 482 patients with RCC from TCGA were used to assess clinical relevance. The Kaplan–Meier method was used to analyze OS using data from the OncoLnc dataset (http://www.oncolnc.org/, accessed on 14 June 2024). This study was conducted in accordance with the standards of the publication guidelines provided by TCGA.

### 4.3. Transfection with siRNAs and shRNAs

A498 and Caki2 cells were transfected with Lipofectamine RNAiMAX transfection reagent (Thermo Fisher Scientific, Waltham, MA, USA) and OptiMEM (Thermo Fisher Scientific) using 50 nM Silencer (Assay ID: 45985; Thermo Fisher Scientific) as *HSF4* siRNA-1 or Stealth siRNA (Assay ID: HSS105061; Thermo Fisher Scientific) as *HSF4* siRNA-2. The negative control was siRNA (D-001810-10; Dharmacon; Horizon Discovery Group plc, Cambridge, UK). For shRNA transfection, we used *Escherichia coli* transfected with shRNA in plasmid DNA (sh-*HSF4*: TRCN0000086281; Sigma-Aldrich, St. Louis, MO, USA). First, *E. coli* cells were incubated in liquid medium with shaking at 37 °C for 12 h. Next, plasmids were extracted using NucleoSpin Plasmid EasyPure (MACHEREY-NAGEL, Dueren, Germany). The extracted plasmids, psPAX2 and pMD2.G (Polysciences, Warrington, PA, USA), were transfected into virus-producing cells (HEK-293T cells) using PEI: Polyethylenimine “Max” (Polysciences). Seventy-two hours later, the virus solution was collected. The recovered virus solution was used to infect Renca cells. The supernatant was removed after 72 h, and new medium containing 0.5 µg/mL puromycin was added to select stably infected cells.

### 4.4. RNA Extraction and RT-qPCR

Three human normal kidney total RNA samples were obtained from Thermo Fisher Scientific (lot nos. 0811001, 0910004, 2055308). mRNA expression levels were measured using a SYBR Green quantitative PCR-based array approach. The *HSF4* primer set used was as follows: forward primer, 5′-GCCTTCCTCGGCAAGCTATG-3′, and reverse primer, 5′-AAACGGCTCTGGTCGCTTAC-3′. The *MET* primer set used was as follows: forward primer, 5′-AGCAATGGGGAGTGTAAAGAGG-3′, and reverse primer, 5′-CCCAGTCTTGTACTCAGCAAC-3′. β-Glucuronidase was used as an endogenous control. The set consisted of a forward primer, 5′-CGTCCCACCTAGAATCTGCT-3′ and a reverse primer, 5′-TTGCTCAAAGGTCACAGG-3′. The specificity of amplification was monitored using a dissociation curve for the amplified product. We used CQ values for relative quantitative evaluation.

### 4.5. Western Blotting

NuPAGE 4–12% Bis-Tris gels (Thermo Fisher Scientific) were used to analyze total protein lysates. The samples were transferred to polyvinylidene difluoride membranes using a Western blot transfer system (Invitrogen, Waltham, MA, USA) according to the manufacturer’s instructions. The antibodies used for immunoblotting were as follows: anti-HSF4 (1:200; cat. no. 18797-1-AP; Proteintech, Rosemont, IL, USA), anti-cleaved caspase 3 (1:250; cat. no. ab2302; Abcam), anti-MET (1:1000; cat. no. 8198; Cell Signaling Technology, Danvers, MA, USA), and anti-β-actin (1:5000; cat. no. bs-0061R; Bioss). Peroxidase-conjugated anti-rabbit IgG (1:5000; cat. no. 7074S; Cell Signaling Technology) was used as the secondary antibody. For quantification, we used ImageJ software (ver. 1.52; https://rsbweb.nih.gov/ij/index.html, accessed on 10 March 2024).

### 4.6. Cell Proliferation, Migration, Invasion, and Tumor Spheroid Formation Assays

Cell proliferation assays were performed using an XTT kit (Roche Diagnostics, Basel, Switzerland). Briefly, RCC cells (1.0 × 10^3^ cells/well) were cultured in 96-well plates. After 96 h, cells were treated with 20 µL XTT reagent and incubated in a 5% CO_2_ incubator at 37 °C. The plate was read 1.5 h later at 450 nm using a microplate reader.

Cell migration was assessed by in vitro wound healing. In experiments using siRNA, cells were seeded at 1 × 10^5^ cells/well in 6-well plates. After incubation for 48 h, cell monolayers were scraped using a P-20 micropipette tip. The difference between the initial gap length after wounding (at 0 h) and the residual gap length (after 18 h) was calculated from micrographs.

Cell invasion ability was examined in 24-well tissue culture plates (BD Biosciences, San Jose, CA, USA) using a modified Boyden chamber consisting of a transwell-precoated Matrigel membrane filter inserted with 8 µm pores. Twenty-four hours later, the number of cells that had passed through the pores and the chamber to adhere to the surface was assessed using micrographs.

Spheroid formation assays were performed to measure cell three-dimensional proliferation ability. RCC cells (3 × 10^4^ cells/well) were cultured in a Cell-able 96-well plate (TOYO GOSEI, Chiba, Japan). After 96 h, random-site micrographs of the spheroids were obtained. To evaluate proliferation ability, the cells were dissolved using a CellTiter-Glo 3D Cell Viability Assay kit (Promega, Madison, WI, USA), and luminescence was evaluated using a TriStar LB941 instrument (Berthold Technologies, Bad Wildbad, Germany).

### 4.7. RNA Sequencing and Gene Enrichment Analysis

A498 and Caki2 cells were seeded at 1 × 10^5^ cells/well in 6-well plates with si-Ctr and si-*HSF4*-1 and 2. After incubation for 72 h, total RNA from RCC cells was extracted by lysing cultured cells in ISOGEN (Nippon Gene, Tokyo, Japan) following the manufacturer’s protocol. Total RNA extracted as described above was subjected to mRNA sequencing (performed by Riken Genesis Corp., Tokyo, Japan). The library was prepared by adding adapters to the fragmented RNA samples. The length of the library was 303–314 bp. Sequencing of the formed clusters in the S4 flow cell was performed using NovaSeq 6000 (Illumina, Inc., San Diego, CA, USA), a next-generation sequencer. The effective read length was 100 bp, and the analysis was performed using the paired or multiplex method. Gene expression data analysis was carried out using the BioPlanet dataset in GeneCodis 4 (https://genecodis.genyo.es/, accessed on 3 July 2024). The gene expression dataset was derived from RNA sequencing.

### 4.8. Apoptosis Assay

For fluorescence-activated cell sorting, cells (1.0 × 10^5^ cells/well) were transfected with siRNA for 72 h as described above. Cells were then collected for subsequent processing. Apoptosis assays were performed by double staining with fluorescein isothiocyanate (FITC)–Annexin V and propidium iodide using a FITC–Annexin V Apoptosis Detection Kit (BD Biosciences, Franklin Lakes, NJ, USA) and a flow cytometer (CytoFLEX Analyzer; Beckman Coulter, Brea, CA, USA). CytExpert 2.4 software (Beckman Coulter) was used to classify the cells into four categories: viable cells, dead cells, early apoptotic cells, and apoptotic cells. Each experiment was repeated at least three times.

### 4.9. Cell Proliferation Assay with Cabozantinib

RCC cells (1.0 × 10^3^ cells/well) were cultured in 96-well plates with si-*HSF4* and cabozantinib (Chem Scene, Monmouth Junction, NJ, USA), which was dissolved in DMSO. IC50 of cabozantinib on SUR-A498 was calculated with IC50 Calculator web tool offered by AAT Bioquest (IC50 Calculator|AAT Bioquest, https://www.aatbio.com/tools/ic50-calculator, accessed on 20 July 2024). After 96 h, cells were lysed using CellTiter-Glo 2D Regent (Promega). Luminescence was evaluated using a TriStar LB941 instrument (Berthold Technologies). The Bliss independence model was used to evaluate the effect of combination therapy. The combined percentage inhibition Y_ab,P_ was calculated as

Y_ab,P_ = Y_a_ + Y_b_ − Y_a_Y_b_

(Y_a_ and Y_b_ are the percentages of inhibited tumor growth).

The observed combined percentage inhibition Y_ab,O_ was then compared with Y_ab,P_. The three scenarios of the efficacy were summarized as

Y_ab,O_ > Y_ab,P_ → Synergy

Y_ab,O_ = Y_ab,P_ → Independent

Y_ab,O_ < Y_ab,P_ → Antagonism

Please refer to the references for the details of calculation formulas [39].

### 4.10. In Vivo Xenograft Model

Animal experiments were approved by the Kagoshima University Animal Experiment Committee (approval no. MD23054) and were conducted in accordance with the animal licensing guidelines of the Kagoshima University Animal Care Committee. Six- to eight-week-old female mice (BALB/c) purchased from CLEA Japan (Tokyo, Japan) were used in the study. The sample size was five or six mice per group, as determined based on the Guidelines for the Welfare and Use of Animals in Cancer Research [40]. We used InVivoMab (Bio X Cell, Lebanon, NH, USA), which targets PD-1, as the ICI for mice at a dose of 5 mg/kg. The ICI was diluted with phosphate-buffered saline (PBS) before intraperitoneal injection. Tumor diameter and mouse weight were measured twice a week. Tumor volume was measured with calipers and calculated as v = (length × width^2^) × (π/6). Fourteen days after inoculation, all mice were euthanized by cervical dislocation, and tumor size was assessed. The excised specimens were embedded in paraffin and used for immunohistochemistry.

### 4.11. Immunohistochemistry

Immunohistochemistry was performed using an UltraVision Detection System (Thermo Fisher Scientific) according to the manufacturer’s instructions. Primary rabbit monoclonal antibodies against Ki67 (cat. no. 12202; Cell Signaling Technology) were diluted at 1:500. The secondary antibody, goat anti-rabbit IgG antibody (H+L), biotinylated (cat. no. BA-1000; Vector Laboratories, San Francisco, CA, USA), was diluted to 5 µg/mL, and incubation was carried out for 30 min. Positive cells were quantified by counting four random microscopic fields. These experimental procedures were described in a previous report [41].

### 4.12. Statistical Analysis

The relationships between two groups were analyzed using Mann–Whitney *U* tests. The relationships between three or more groups were analyzed using the multiple comparison test with the Bonferroni/Dunn method. The effect sizes were analyzed by calculating Cliff’s delta. All analyses were performed using Expert Statview software, version 5.0 (SAS Institute, Inc., Cary, NC, USA) or R programming language version 4.4.2 (R Core Team).

## Data Availability

All the data generated or analyzed during this study are included in this published article.

## Supplementary Materials

The following supporting information can be downloaded at: https://www.mdpi.com/article/10.3390/ijms26041776/s1.

## Abbreviations

The following abbreviations are used in this manuscript:

HSF4	heat shock transcription factor 4
RCC	renal cell carcinoma
ICI	immune checkpoint inhibitor
TCGA	The Cancer Genome Atlas
RNAi	RNA interference
